# Correspondence

**Published:** 1859-11

**Authors:** N. B. Slayton


*Madison, Oct.* 5, 1859.

MESSRS. EDITORS:—In reply to Dr. Hunter, I shall use no Billingsgate slang. The term *lie* sounds to me rather unprofessional, and I hope, for the credit of the profession, as Dr. Hunter is somewhat *noted*, it may be set down as a slip of the pen; if intentional, it shows a *bad* spirit, or a desperate cause Remember, Doctor, "Whom the gods destroy, they first make mad." Now, keep cool, for you seem to be simply endeavoring to protect the profession from imposition, and too much zeal might leave the supposition of *monomania*, to say the least.

My dear Doctor, I was well aware you could obtain my specifications, and I entertained no doubt but that you would do so, when I penned my article. You have appealed to the documents, and to the documents let us go.

Your copy from the specifications is correct, and from that you will see that I make but two claims, *one for mounting teeth*, the other in *forming the rim.*

My patent reads : I " take gold or silver, or both combined, *properly prepared*, *by precipitation or other modes*, *with mercury.*" I do not specify how I prepare my amalgam. Success depends entirely upon this point, it being *properly* prepared. How *is* it prepared? Dr. Hunter has not told us. Whoever attempts to work amalgam as he has described will have his labor for his pains. It can be prepared but *one* way, while the manipulations may be performed in various ways, without invalidating the patent.

In my specifications I simply state that I use gold or silver *properly* prepared, leaving just enough untold to defeat just such efforts as you are now making. Can you pardon me, Doctor, for mistifying you a little? I admit that 'tis great "*impudence*" to dare know anything or do anything not already done by Dr. William M. Hunter.

You seem to be working for the interest of the profession, and your disinterested efforts will be *duly appreciated* by those who know you *best.*

If my work is such a "*worthless humbug*" as the Doctor describes, let it be shown in such a manner as to remove all doubt, and then let it be condemned by all—a consummation to be wished by you, I doubt not. To further the exposition of its true character, and place the whole matter in the right light, I now make you two propositions :

First, That we each make an upper set of teeth on silver base, using teeth of our own selection, of Jones & White's manufacture.

You make yours as you are in the habit of making for your patients—the work to be done in the best manner you or all your workmen combined are capable of doing. You shall use no amalgam, *soft solder or tin.* I will make mine as I am in the habit of making for my patients, as I instruct those I sell to, and in a manner covered by my patents. I will flow no solder through the amalgam, to make it more dense.

At the appointed time, which may be designated by yourself, both pieces to be placed in the hands of Dr. Wardell, of Cincinnati, or such other person as he may agree upon, subject to inspection for the space of four weeks by all dentists who may be pleased to call, each giving his opinion of the two pieces, in point of *strength,* beauty of construction, cleanliness, neatness, and every other point calculated to make a perfect piece of work.

Neither piece to be marred or injured in any way, and neither you, nor I, nor your partners to see the other's works. The strength shall be tested by Dr. Wardell or his substitute, should this selection be made.

If the majority of the dentists pronounce my work the best in the features mentioned, I shall take both sets, you to acknowledge in as public a manner as your attack, that your charges are unjust.

If, on the other hand, your charges against my work are sustained by the majority of dentists examining the two pieces, you shall take both pieces, I will throw up my patent, and publish to the world.

If you object to this test being submitted to the dentists of Cincinnati and such others that may call to examine them, the pieces may be sent to either of Jones & White's dental depots, and submitted to the judgment of the dentist of the city to which they may be sent.

I specify a silver base, simply because it is less expensive, but I think gold works much better.

Now about its cleanliness. You say in your first article that it admits the fluids of the mouth, becomes *putrid, offensive,* and *filthy, beyond description.* To test this point, I propose that you and myself procure each a set of teeth on gold base, worn not less than three months, from some one of our patients, whose testimony can be relied upon; your piece made as you make gold plates, mine to be amalgam, both pieces to be taken from the mouth, and conveyed to Dr. Wardell or his substitute, without any cleaning whatever, in such way as to be satisfactory to both parties that the thing is fairly done. He shall cleanse them in simple soap and water, and then remain in his hands for one week for exhibition, that the absolute cleanliness of the two pieces may be determined.

Neither of the two pieces shall, in any manner, be injured, strained or defaced in any way, but returned to their respective owners in the same condition as when procured. The result of this second practical test to be considered in making up the decision as to the general merits of the work.

Such appears to me to be the proper way of settling a professional controversy, and doubtless will be satisfactory to the profession at large.

Now, Doctor, you have my *proposition* before you. If you are a *man*, and honest in professed intentions, you will accept. If you sustain your position, you will have the honor of exposing a great humbug.

If you decline, your true position can be understood by the profession, and the "*Yahoos*" may have the pleasure of holding you up to public gaze in your true character, and *perhaps* I may say something that you will wish had not been said.

N. B. SLAYTON.

